# Effectiveness and Safety of Different Patch Materials for Supravalvar Aortic Stenosis (Middle-Term Outcomes)

**DOI:** 10.31083/j.rcm2501014

**Published:** 2024-01-09

**Authors:** Xinyue Lang, Lizhi Lv, Simeng Zhang, Aihua Zhi, Cheng Wang, Qiang Wang

**Affiliations:** ^1^Medical Research & Biometrics Center, National Center for Cardiovascular Diseases, The National Clinical Research Center for Cardiovascular Diseases, Fuwai Hospital, Chinese Academy of Medical Sciences & Peking Union Medical College, 102300 Beijing, China; ^2^Department of Pediatric Cardiac Center, Beijing Anzhen Hospital, Capital Medical University, 100029 Beijing, China; ^3^Department of Cardiac Surgery, Yunnan Fuwai Cardiovascular Hospital, 650102 Kunming, Yunnan, China; ^4^Department of Cardiac Surgery, Peking University People’s Hospital, 100044 Beijing, China; ^5^Department of Radiology, Fuwai Hospital, National Center for Cardiovascular Diseases, Chinese Academy of Medical Sciences and Peking Union Medical College, 100037 Beijing, China; ^6^Department of Radiology, Yunnan Fuwai Cardiovascular Hospital, 650102 Kunming, Yunnan, China; ^7^Center for Pediatric Cardiac Surgery, Fuwai Hospital, National Center for Cardiovascular Diseases, Chinese Academy of Medical Sciences and Peking Union Medical College, 100037 Beijing, China

**Keywords:** supravalvular aortic stenosis, surgical repair, pericardium patch, modified patch, artificial patch

## Abstract

**Background:**

To determine the effectiveness and safety of different patch materials in the treatment of pediatric patients with congenital supravalvular aortic stenosis (SVAS).

**Methods:**

218 consecutive SVAS patients (age <14 years) who underwent surgery from Beijing Fuwai and Yunnan Fuwai hospital between 2002 and 2020 were included. Patients were divided into the pericardium patch group (133 (61.0%)), modified patch group (43 (19.7%)) and artificial patch group (42 (19.3%)). The primary safety endpoint was patch-related adverse complications (post-operation patch hemorrhage or aortic sinus aneurysm at 2-year follow-up). The primary effectiveness outcome was the re-operation or restenosis at 2-year follow-up. Multivariable cox regression was used to obtain the hazard ratio (HR).

**Results:**

The median age at operation was 43.5 months (IQR 24.0–73.0). Only three patients had patch-related adverse complications, and no difference existed among the three groups (*p* = 0.763). After a median follow-up of 24.0 months (IQR 6.0–48.0), patients with a pericardium patch had a lower re-operation or restenosis rate compared with the other two groups (pericardium patch vs modified patch, HR = 0.30, 95% CI 0.12–0.77; pericardium patch vs artificial patch, HR = 0.33, 95% CI 0.13–0.82), even in the main subgroup and sensitivity analysis.

**Conclusions:**

In pediatric patients, the safety of autologous pericardium patch is acceptable, along with lower rates of middle-term re-operation or restenosis.

**Clinical Trial Registration:**

http://www.chictr.org.cn, number: ChiCTR2300067851.

## 1. Introduction

Congenital supravalvular aortic stenosis (SVAS) is the rarest form of obstruction of the left ventricular outflow tract, accounting for less than 0.05% of all congenital heart defects [1]. The malformation is typically characterized by hourglass-shaped narrowing of the aorta at the sinotubular junction (STJ) and, in some cases, the narrowing of the entire ascending aorta and arcuate branches [2]. Early intervention is essential in adolescents because the progressive nature of the stenosis increases the risk of sudden death [3, 4, 5, 6].

The first successful surgical correction of SVAS was reported in 1961 [7]. Since then, a variety of operative techniques emerged, differing by the number of Valsalva sinuses which was augmented by (patch) repair. Along with the improvement of surgical procedures, the variety of patch materials was also increasing. Magoon used a compressed polyvinyl sponge as patch material for the first time to widen STJ [7]. Subsequently, researchers found that only one patch was not sufficient to widen the STJ and proposed the use of polyester fabric as a patch material based on improving the number of its patches [8]. Considering the risk of aortic regurgitation in the distant postoperative period, some investigators proposed the application of prosthetic material (autologous pericardium) for symmetrical triple patch placement in 1988 [9]. However, some researchers considered that autologous pericardium could not withstand the blood flow pressure and would dilate into an aortic sinus aneurysm [10], so modified autologous pericardial techniques such as glutaraldehyde-treated pericardium and outer lining with other material have been designed to increase pressure resistance.

The early treatment of SVAS is satisfactory, but the high rate of restenosis and re-operation in the distant future remains a major concern [11, 12, 13, 14]. Currently, studies have focused on the differences in the efficacy of different surgical procedures for the treatment of SVAS. However, it is unknown which patch materials have a better prognosis. Thus, this study aims to review our center’s experience using pericardium patches, modified patches, and artificial patches for the treatment of congenital SVAS.

## 2. Materials and Methods

### 2.1 Patient Population

This retrospective cohort study included consecutive patients in Beijing Fuwai hospital and Yunnan Fuwai hospital from March 2002 to April 2020. Eligible patients were younger than 14 years old and had congenital SVAS undergoing surgical repair. The diagnosis of SVAS was documented by a trans-thoracic echocardiogram (TTE). Patients without patch implantation were excluded. The study protocol conforms to the ethical guidelines of the 1975 Declaration of Helsinki as reflected in a priori approval by the institution’s human research committee (no.2021-1578). Informed consent was waived for retrospective collection and analysis of deidentified demographic and medical data. This study was registered at the Chinese Clinical Trial Registry (https://www.chictr.org.cn/), ChiCTR2300067851, accessed on 2023.01.29.

### 2.2 Patch Materials

**Fig. 1.**
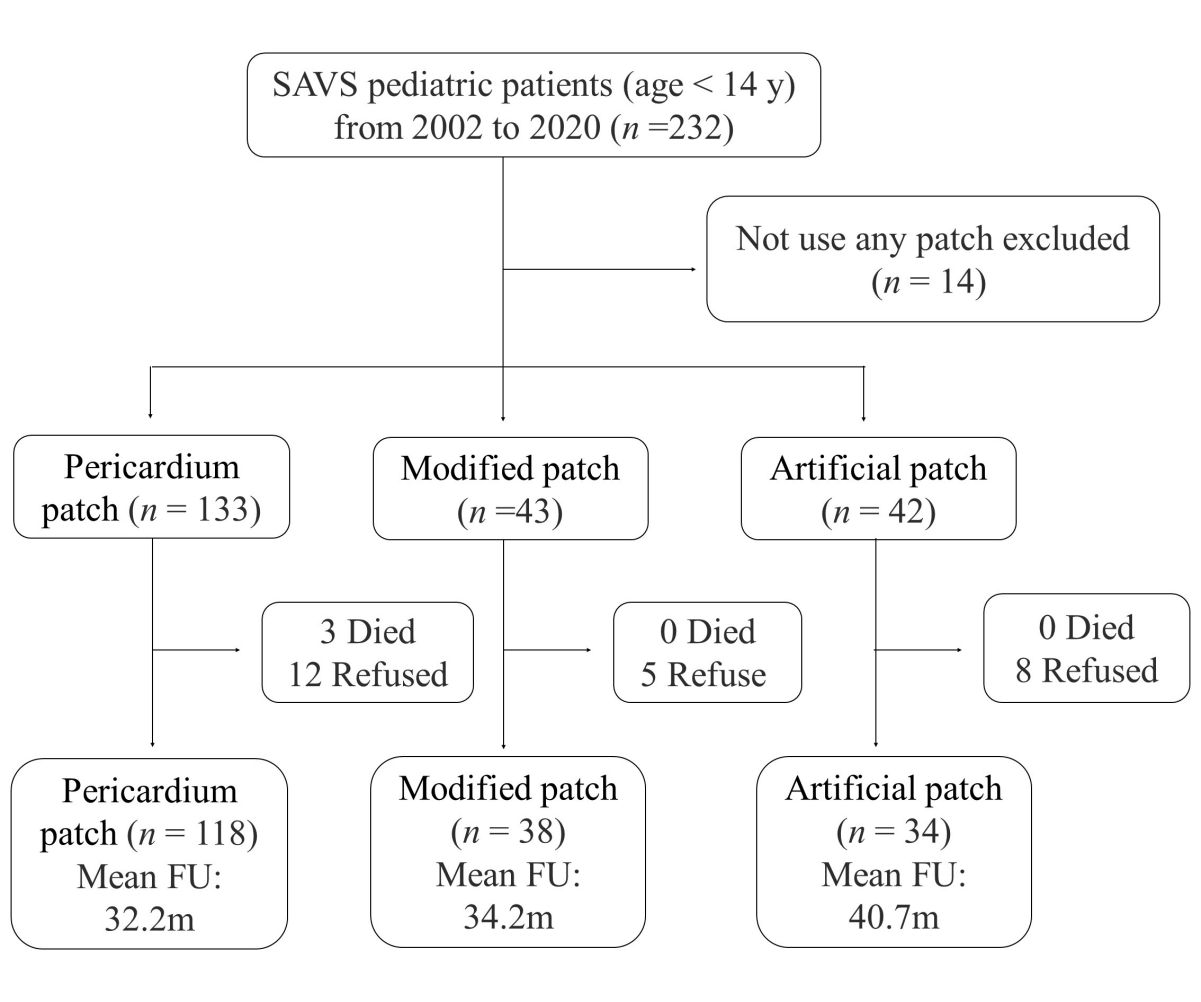
**Flow chart of patient selection and follow-up**. Abbreviation: FU, follow up; SVAS, supravalvular aortic stenosis.

We separated the patch material used for the first surgical correction of SVAS into three groups, pericardium patch, modified patch, and artificial patch (Fig. 1). The pericardium patch group (n = 133) was untreated fresh autologous pericardium. The modified patch (n = 43) included glutaraldehyde-treated autologous pericardium, Bovine pericardium™ (JHZB Biotech group, Zhejiang, China), and both together. The glutaraldehyde-treated autologous pericardium was prepared by soaking the pericardium in 0.6% glutaraldehyde for 10 min. The artificial patch group (n = 42) were Dacron™ (Maquet Getinge Group, Rastatt, Germany) and Artificial vascular patch™ (W. L. Gore & Associate, LLC, AZ, USA).

### 2.3 Surgical Technique

All patients underwent median sternotomy, a cardiopulmonary bypass with bicaval cannulation, and left ventricular venting through the right upper pulmonary vein. The HTK® cardioplegia (CUSTODIOL, Barcelona, Spain) was used for myocardial protection.

In the single-patch method (McGoon repair), a teardrop-shaped patch was used for the aortic root augmentation after longitudinal incision through the stenotic site extending to the non-coronary sinus. The two-patch method (Doty repair) involved a pantaloon-shaped patch plasty. The three-patch method (Brom repair) enlarged the aortic root into three aortic sinuses with three separate “Shield”-shaped patches (**Supplementary Fig. 1**). Other concomitant cardiovascular anomalies were treated at the same time.

### 2.4 Variables and Outcomes

Baseline information, echocardiographic data, pre-, intra-, post-operative, and follow-up data were obtained from cardiac surgery databases. The z-score of aortic valves, STJ, and ascending aorta were calculated according to the Boston Children’s Hospital echocardiography calculation tool (https://zscore.chboston.org/).

The primary safety endpoint was patch-related adverse complications (post-operation patch hemorrhage or aortic sinus aneurysm at 2-year follow-up). The primary effectiveness outcome was re-operation or restenosis (defined as peak supravalvar aortic gradients over 40 mmHg [15]) at 2-year follow-up.

Secondary outcomes included re-operation, restenosis, left ventricular ejection function (LVEF), supravalvar aortic gradients, aortic valve z-score, STJ z-score, ascending aorta z-score, and aortic valve regurgitation at follow-up.

### 2.5 Data Analysis

Continuous variables were reported as mean (standard deviance, SD) and median (inter-quartile range, IQR). Dichotomous variables were reported as the frequency (percentage). Analysis of variance was used to compare normally continuous variables and the Kruskal-Wallis H test was to compare non-normally distributed continuous variables. The Pearson chi-squared test or Fisher’s exact test was used to compare categorical data. For dichotomous outcomes, odds ratios (OR) were calculated using logistic regression models. For continuous outcomes, βcoefficient was calculated using linear regression models. Kaplan–Meier plot was used to depict the cumulative events of the primary effectiveness outcome and stratified according to the patch materials. Hazard ratios (HR) were calculated using Cox proportional hazards regression models. Subgroup analyses for age (<7 or ≥7-years-old), gender, surgical technique (single-patch or two-patch), and operators (experienced or inexperienced) were conducted. To avoid confounding bias, the regression models were adjusted for age, gender, concomitant cardiovascular anomaly, surgical technique, and pre-operation transvalvular pressure gradient based on clinical experience and baseline balance among groups. Sensitivity analyses were conducted using the Inverse Probability Treatment Weighting (IPTW) method to test the robustness of the primary analysis. The propensity score was calculated based on age, gender, supravalvar aortic gradients, pulmonary valve stenosis, bicuspid aortic valve, patent ductus arteriosus, and ventricular septal defect. The variable selection for propensity score was considered by clinical experience and baseline balance among groups. The stepwise method was used to determine the variable in the multivariable logistic regression models. Missing data were imputed using multiple imputation methods. A two-sided *p*-value < 0.05 was considered to be significant for the comparisons among the three groups. For the multiple comparisons, the Bonferroni correction was used, and a two-sided *p*-value < 0.025 was considered to be significant. All analyses were conducted using R (version 4.0.3, AT&T, Auckland, New Zealand).

## 3. Results

### 3.1 Baseline Information

Among 218 pediatric patients, 133 (61.0%) used a pericardium patch, 43 (19.7%) used a modified patch and 42 (19.3%) used an artificial patch. The median age at operation was 43.5 months (IQR: 24.0–73.0). Patients using modified patches (median: 64.0, IQR: 36.0–96.0) or artificial patches (median: 53.0, IQR: 33.0–85.0) were older than patients using pericardium patches (median: 36.0, IQR: 21.0–61.0). Less than 40% of patients were female in each group. There was no difference in echocardiographic information among the three groups. Patients using pericardium patches had more pre-operative concomitant cardiovascular anomalies compared with other patches (Table 1, **Supplementary Table 1**). Detailed information on patch materials used and proportions in previous years was shown in **Supplementary Fig. 2**. The overall trend suggested that as the number of patients treated per year increases, the median age of patients decreases over the past few years.

**Table 1.** **Baseline characteristics and inro-operative information of patients with SVAS**.

Variables		Pericardium patch (n = 133)	Modified patch (n = 43)	Artificial patch (n = 42)	*p* value
Age (months)		43.9 ± 32.5	63.4 ± 35.6	61.4 ± 35.9	0.001
		36.0 (21.0, 61.0)	64.0 (36.0, 96.0)	53.0 (33.0, 85.0)	
Women		46 (34.6)	13 (30.2)	10 (23.8)	0.414
BSA ($m^{2}$)		0.6 ± 0.2	0.7 ± 0.2	0.8 ± 0.4	0.001
		0.6 (0.5, 0.7)	0.7 (0.5, 0.9)	0.7 (0.5, 1.0)	
Diameter of the stenosis (mm)		7.3 ± 2.2	7.1 ± 1.9	7.9 ± 2.2	0.196
		7.0 (6.0, 8.2)	7.0 (6.0, 8.0)	7.8 (6.0, 10.0)	
Aortic valve z-score		0.2 ± 1.3	0.1 ± 1.4	–0.0 ± 1.3	0.556
		0.1 (–0.6, 0.8)	0.1 (–0.7, 1.1)	0.1 (–0.8, 0.7)	
STJ z-score		1.9 ± 1.6	1.7 ± 1.7	1.5 ± 1.4	0.391
		1.9 (1.0, 2.7)	1.9 (0.4, 2.9)	1.3 (0.6, 1.9)	
Ascending aorta z-score		–0.9 ± 1.7	–1.1 ± 1.6	–1.1 ± 1.6	0.846
		–1.1 (–2.2, –0.1)	–1.1 (–2.1, –0.4)	–1.3 (–2.1, –0.6)	
Type II		9 (6.8)	1 (2.3)	4 (9.5)	0.387
Concomitant cardiovascular ${\mathrm{anomaly}}^{a}$		56 (42.1)	12 (27.9)	11 (26.2)	0.078
PS		38 (28.6)	10 (23.3)	4 (9.5)	0.041
PVS		6 (4.5)	1 (2.3)	2 (4.8)	0.801
Bicuspid aortic valve		16 (12.0)	2 (4.7)	6 (14.3)	0.305
Inro-operative					
Surgical technique					0.053
	Single-patch	73 (54.9)	22 (51.2)	15 (35.7)	
	Two-patch	52 (39.1)	21 (48.8)	26 (61.9)	
	Three-patch	8 (6.0)	0 (0.0)	1 (2.4)	
${\mathrm{Operators}}^{b}$					0.050
	Experienced	72 (54.1)	14 (32.6)	20 (47.6)	
	Inexperienced	61 (45.9)	29 (67.4)	22 (52.4)	
CPB (min)		111.9 ± 67.9	113.7 ± 70.4	96.8 ± 39.5	0.366
		91.0 (74.0, 122.0)	95.0 (80.0, 120.0)	88.5 (73.0, 100.0)	
CCP (min)		70.4 ± 37.4	70.7 ± 29.8	60.7 ± 19.9	0.237
		60.0 (47.0, 84.0)	58.0 (52.0, 83.0)	56.5 (45.0, 72.0)	

^a^Concomitant cardiovascular anomaly means patients had PS, PVS or bicuspid aortic valve. ^b^Experienced operators were defined as having completed more than 20 operations. Abbreviation: BSA, body surface area; CPB, cardiopulmonary bypass; CCP, cross-clamping; PS, pulmonary stenosis; PVS, pulmonary valve stenosis; STJ, sinotubular junction; SVAS, supravalvular aortic stenosis.

### 3.2 Operative and Postoperative Information

The application of surgical techniques was different among the three groups (*p* = 0.026). The single-patch method was used most in the pericardium patches (73 (54.9%)) compared with the modified (22 (51.2%)) and the artificial patches (15 (35.7%)). For the operators’ experience, cardiopulmonary bypass time, and cross-clamping time, there was no significant difference between the three groups (Table 1).

Three patients (1.4%) died in the hospital and no difference existed among the three groups (*p* = 0.345). All dead patients were treated with the single-patch method, two of them were caused by heart failure and the remaining one was caused by pulmonary arterial hypertension. No significant difference was observed in surgery-related complications and echocardiographic information among the three groups. Each of the pericardial and modified patch groups had one patient with post-operative patch hemorrhage. During the hospitalization, ten patients had re-operation (4.5%, 7.0%, and 2.4%, separately). One patient underwent a re-correction of SVAS, two patients underwent diaphragmatic plication, two patients underwent surgical hemostasis, four patients underwent chest closure, and one patient underwent subaortic membrane resection (Table 2).

**Table 2.** **Post-operative information of patients with SVAS**.

Variables		Pericardium patch (n = 133)	Modified patch (n = 43)	Artificial patch (n = 42)	*p* value	β/OR ${\mathrm{(95\% CI)}}^{a}$	
						Pericardium patch vs Modified patch	Pericardium patch vs Artificial patch
Surgery-related complications							
	Patch hemorrhage	1 (0.8)	1 (2.3)	0 (0.0)	NA	NA	NA
	Repeated aortic clamping	6 (4.5)	3 (7.0)	0 (0.0)	0.254	0.37 (0.08, 1.69)	NA
						*p* = 0.198	
	Cardiac defibrillation	13 (9.8)	4 (9.3)	7 (16.7)	0.426	1.19 (0.35, 4.04)	0.66 (0.23, 1.84)
						*p* = 0.786	*p* = 0.423
	AMI	1 (0.8)	0 (0.0)	0 (0.0)	0.725	NA	NA
	Arrhythmia	2 (1.5)	0 (0.0)	0 (0.0)	0.525	NA	NA
Post-operation							
	LVEF	66.6 ± 6.4	66.6 ± 8.2	68.5 ± 6.1	0.265	0.30 (–2.11, 2.70)	–1.50 (–3.92, 0.92)
		65.0 (62.0, 70.0)	66.0 (64.0, 72.0)	66.5 (65.0, 72.0)		*p* = 0.809	*p* = 0.225
	Aortic valve z-score	0.4 ± 1.7	–0.1 ± 1.4	–0.1 ± 1.6	0.096	0.37 (–0.14, 0.88)	0.22 (–0.29, 0.74)
		0.2 (–0.7, 1.2)	–0.4 (–1.0, 0.7)	–0.3 (–1.2, 1.1)		*p* = 0.157	*p* = 0.399
	STJ z-score	1.7 ± 2.2	1.5 ± 1.8	1.2 ± 1.9	0.393	0.09 (–0.59, 0.77)	0.19 (–0.50, 0.89)
		1.4 (0.3, 2.5)	1.2 (0.0, 2.6)	1.0 (–0.5, 2.6)		*p* = 0.798	*p* = 0.582
	Ascending aorta z-score	–0.4 ± 1.9	–0.8 ± 1.5	–0.9 ± 1.5	0.269	0.28 (–0.33, 0.89)	0.27 (–0.35, 0.89)
		–0.7 (–1.5, 0.3)	–1.1 (–1.8, 0.2)	–1.1 (–2.0, 0.3)		*p* = 0.374	*p* = 0.395
	Re-operation during hospitalization	6 (4.5)	3 (7.0)	1 (2.4)	0.598	0.43 (0.09, 1.93)	1.45 (0.16, 13.24)
						*p* = 0.268	*p* = 0.742
	Death	3 (2.3)	0 (0.0)	0 (0.0)	0.345	NA	NA

^a^The models were adjusted for age, gender, concomitant cardiovascular anomaly, surgical technique, and pre-operation supravalvar aortic gradients. Abbreviation: AMI, acute myocardial ischemia; CI, confidence interval; LVEF, left ventricular ejection fraction; OR, odds ratio; STJ, sinotubular junction; SVAS, supravalvular aortic stenosis; NA, not available.

### 3.3 Follow-Up Outcomes

The echocardiographic follow-up was conducted in 89.2% (190/213) of patients. The baseline information of patients with follow-up did not differ from patients without follow-up. The median follow-up duration was 24.0 months (IQR: 6.0–48.0).

At follow-up, no death occurred and eight patients (4.2%) had re-operation. Four patients underwent re-correction of SVAS, two of whom underwent aortic arch surgery and aortic valvuloplasty at the same time, two patients underwent Ross surgery, and two patients underwent aortic valvuloplasty.

Aortic sinus aneurysm was only found in one case (0.8%) in the pericardium patch group during the follow-up, for the primary safety outcome, two (1.7%) patients had patch-related adverse complications in the pericardium patch group, one (2.6%) patient had patch-related adverse complications in the modified patch group, and no patients in the artificial patch group. No difference was found between the three groups (*p* = 0.763).

**Fig. 2.**
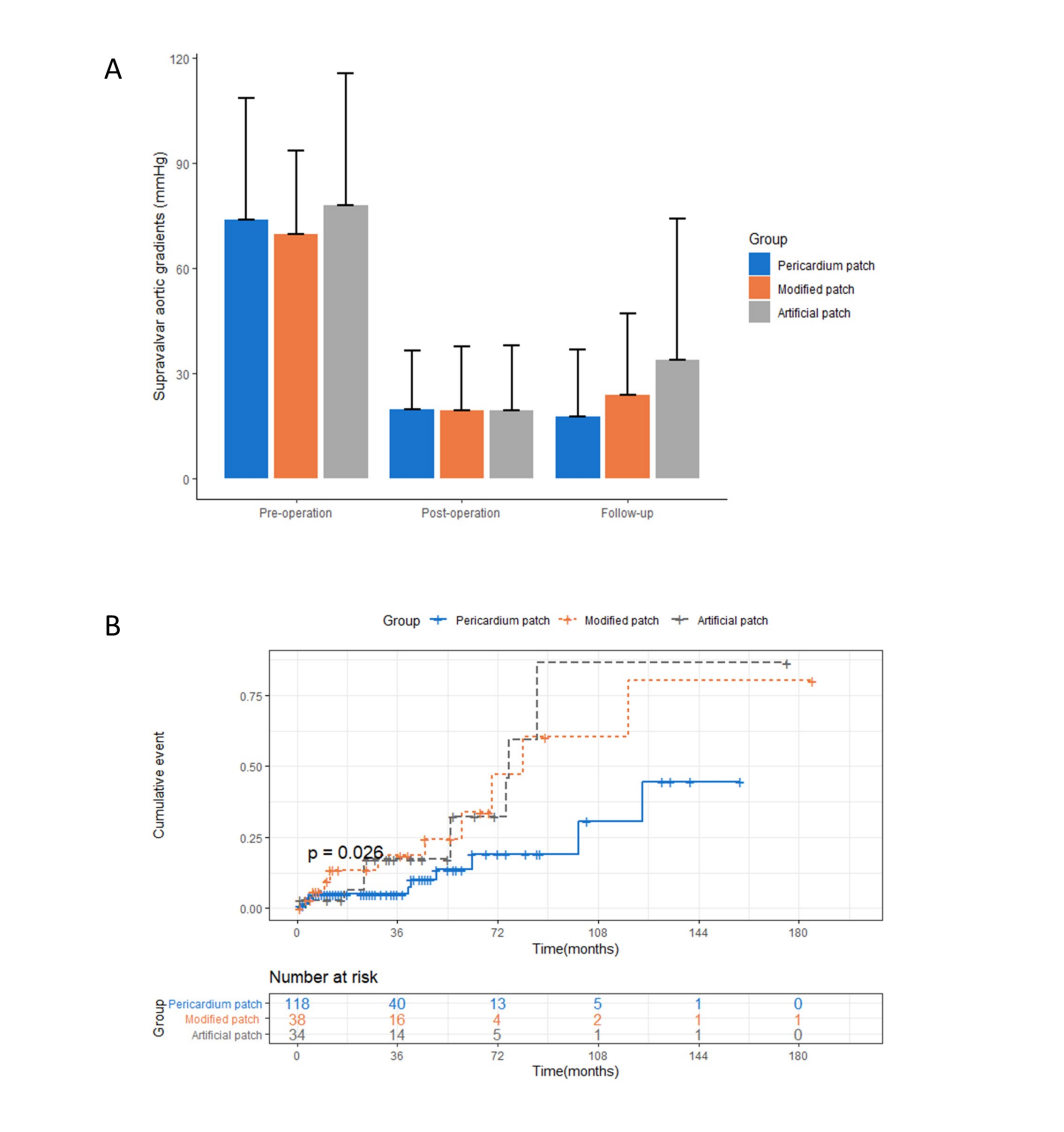
**Outcomes of the pericardium patch, modified patch, and artificial patch**. (A) Transvalvular pressure gradient at baseline, post-operation, and follow-up stratified by patch material. The difference in follow-up transvalvular pressure gradient among groups was found by the Anova test (*p* < 0.05). (B) Cumulative events of the primary effectiveness outcome by Kaplan–Meier plot. The difference among groups was found by the log-rank test (*p* < 0.05).

For the primary effectiveness outcome, the pericardium patches performed better, with a lower composite outcome rate (re-operative or restenosis at 2-year follow-up) of 9.3% compared with the modified patches (26.3%) and the artificial patches (32.4%). And the primary effectiveness outcome was stable after adjusting for age, gender, concomitant cardiovascular anomaly, surgical technique, and pre-operation supravalvar aortic gradient (pericardium patch vs modified patch, OR = 0.29, 95% CI 0.10 to 0.78, *p* = 0.015; pericardium patch vs artificial patch, OR = 0.28, 95% CI 0.11 to 0.72, *p* = 0.008). The follow-up supravalvar aortic gradient of the pericardium patches (17.8 ± 18.9) did not increase compared with post-operation (19.8 ± 16.9), while it increased slightly for the modified patches and significantly for the artificial patches (Table 3, Fig. 2A). The pericardium patches (0.2 ± 2.1) had a higher ascending aorta z-score compared with the modified patches (–0.8 ± 2.5) and artificial patches (–1.0 ± 2.2).

**Table 3.** **Two-year follow-up characteristics of patients with SVAS**.

Variables		Pericardium patch (n = 118)	Modified patch (n = 38)	Artificial patch (n = 34)	*p* value	β/OR ${\mathrm{(95\% CI)}}^{a}$	
						Pericardium patch vs Modified patch	Pericardium patch vs Artificial patch
Primary effectiveness outcome							
	Composite ${\mathrm{outcome}}^{b}$	11 (9.3)	10 (26.3)	11 (32.4)	0.002	0.29 (0.10, 0.78)	0.28 (0.11, 0.72)
						*p* = 0.015	*p* = 0.008
Primary safety outcome							
	Patch-related adverse complications	2 (1.7)	1 (2.6)	0 (0.0)	0.763	NA	NA
Secondary outcome							
	Re-operation at 2 y follow-up	3 (2.3)	2 (4.7)	3 (7.1)	0.316	0.18 (0.02, 1.40)	0.20 (0.03, 1.16)
						*p* = 0.102	*p* = 0.073
	Restenosis at 2 y follow-up	9 (7.6)	9 (23.7)	9 (26.5)	0.004	0.32 (0.11, 0.92)	0.29 (0.10, 0.85)
						*p* = 0.035	*p* = 0.023
	LVEF	67.8 ± 4.7	66.8 ± 5.9	65.8 ± 3.7	0.130	0.52 (–1.41, 2.45)	2.10 (0.16, 4.03)
		67.2 (65.0, 72.0)	66.5 (63.0, 70.0)	66.0 (64.0, 68.0)		*p* = 0.598	*p* = 0.035
	Aortic valve z-score	–0.0 ± 1.6	–0.1 ± 2.1	–0.3 ± 1.8	0.714	0.44 (–0.23, 1.11)	0.26 (*–*0.40, 0.93)
		–0.2 (–0.9, 0.7)	–0.3 (–1.4, 0.7)	–0.3 (–1.7, 0.6)		*p* = 0.198	*p* = 0.440
	STJ z-score	2.0 ± 2.3	1.5 ± 2.0	1.4 ± 2.5	0.296	0.30 (*–*0.61, 1.21)	0.38 (*–*0.53, 1.29)
		1.6 (0.3, 3.0)	1.4 (0.1, 2.9)	0.9 (–0.5, 2.8)		*p* = 0.523	*p* = 0.413
	Ascending aorta z-score	0.2 ± 2.1	–0.8 ± 2.5	–1.0 ± 2.2	0.003	1.03 (0.15, 1.90)	1.13 (0.25, 2.01)
		–0.0 (–1.2, 1.4)	–1.3 (–2.2, 0.7)	–1.5 (–2.4, 0.1)		*p* = 0.023	*p* = 0.013
	Aortic valve regurgitation	5 (3.8)	4 (9.3)	5 (11.9)	0.119	0.56 (0.12, 2.57)	0.39 (0.11, 1.41)
						*p* = 0.457	*p* = 0.150
	Aortic sinus aneurysm	1 (0.8)	(0.0)	(0.0)	1.000	NA	NA

^a^The models were adjusted for age, gender, concomitant cardiovascular anomaly, surgical technique, and pre-operation supravalvar aortic gradients. ^b^Composite outcome was defined as re-operation or restenosis at 2 y follow-up. Abbreviation: CI, confidence interval; LVEF, left ventricular ejection fraction; OR, odds ratio; STJ, sinotubular junction; SVAS, supravalvular aortic stenosis; NA, not available.

**Fig. 3.**
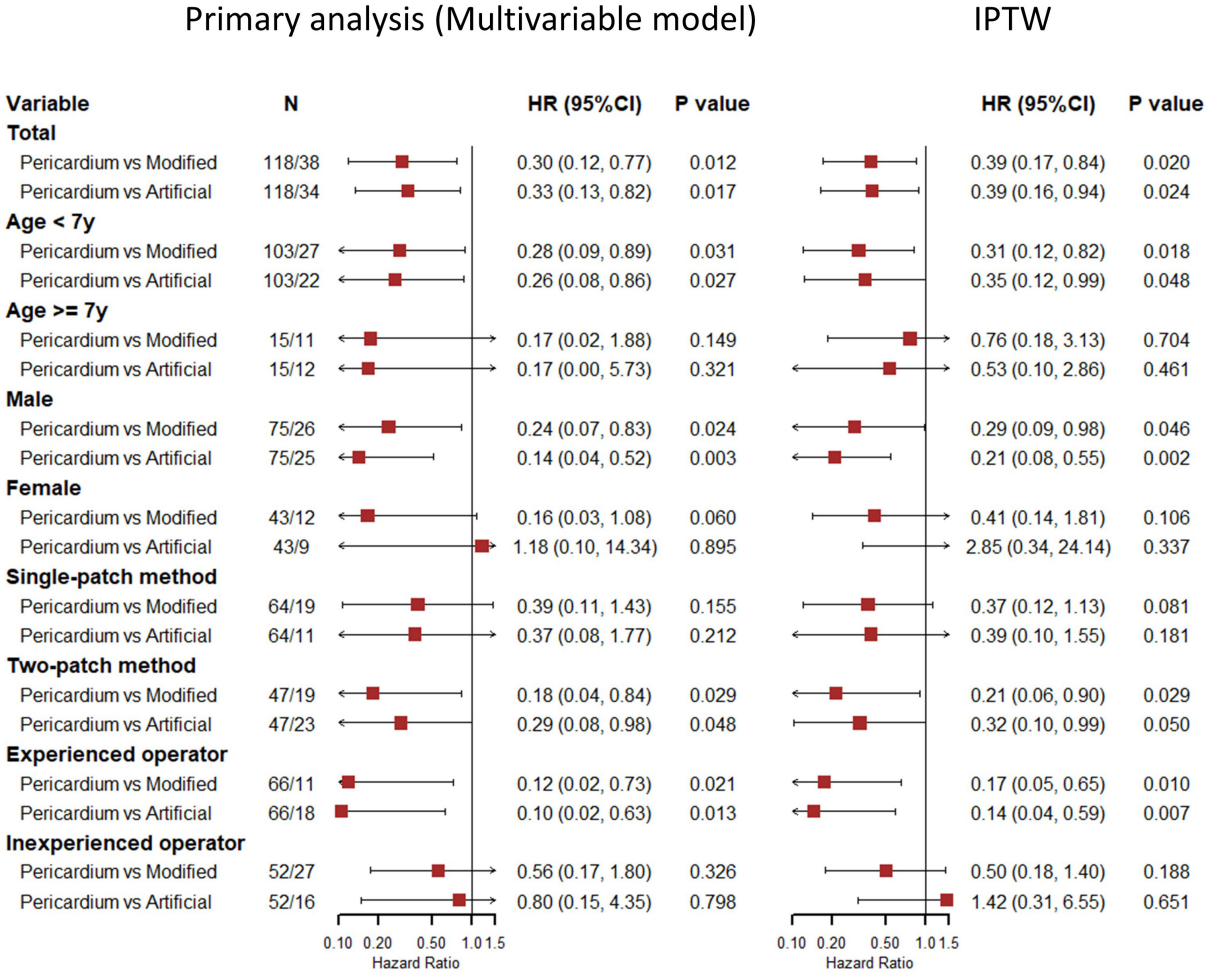
**Subgroup and sensitivity analyses of the primary effectiveness outcome**. Pericardium means pericardium patch, modified means modified patch, and artificial means artificial patch. For the primary analysis, the models were adjusted for age, gender, concomitant cardiovascular anomaly, surgical technique and pre-operation supravalvar aortic gradients. And the subgroup models excluded the variate itself as the covariate. The sensitivity analyses were conducted using IPTW method. Abbreviation: CI, confidence interval; HR, hazard ratio; IPTW, inverse Probability Treatment Weighting.

Kaplan–Meier survival curves for the primary effectiveness outcome are shown in Fig. 2B. After adjusting for age, gender, concomitant cardiovascular anomaly, surgical technique, and pre-operation supravalvar aortic gradient, the pericardium patches had a lower re-operative or restenosis rate compared with the modified patches and the artificial patches during the follow-up period (pericardium patch vs modified patch, HR = 0.30, 95% CI 0.12 to 0.77, *p* < 0.012; pericardium patch vs artificial patch, HR = 0.33, 95% CI 0.13 to 0.82, *p* < 0.017). Subgroup analysis for the primary effectiveness outcome gave a similar result for the age <7 years, male, two-patch method, and experienced operator group. Sensitivity analyses were conducted using the IPTW method proving the robustness of the primary analysis (Fig. 3).

## 4. Discussion

### 4.1 Main Findings

We summarized the patch application in Beijing Fuwai hospital and Yunnan Fuwai hospital over the past 20 years. The results demonstrated that the pericardium patch used for SVAS treatment in adolescents <14 years had better effectiveness outcomes (lower re-operative and restenosis rates) with guaranteed safety compared with the modified patch and artificial patch. Meanwhile, subgroup analysis for the age <7 years, male, two-patch method, experienced operator group, and sensitivity analyses also indicated the superiority of the pericardium patch.

### 4.2 Safety Assessment

In relation to the safety assessment, some researchers were concerned that the autologous pericardium lacked tissue strength and could increase the risk of hemorrhage from patch rupture or the risk of aortic dilatation to form an aortic sinus aneurysm [16, 17]. We found only one case of patch hemorrhage and one case of aortic sinus aneurysm (0.8%, respectively), which indicated that the pericardium patch could ensure long-term patency and had a low risk of adverse aortic dilatation in the postoperative period and during follow-up. In addition, the previous study has proven the toughness of the autologous pericardium to withstand the flow pressure of the aortic root in adults [18]. The mean follow-up time for the application of autologous pericardium in our center is currently 32.2 months, and we will continue to follow this cohort to observe the long-term condition of the aortic root.

Three patients (1.4%) using pericardium patches died during hospitalization, but they had more preoperative concomitant cardiovascular anomalies (pulmonary stenosis, pulmonary valve stenosis (PVS), and bicuspid aortic valve) and their preoperative pressure gradients were more severe compared with other two groups. Previous studies indicated that preoperative combined pulmonary stenosis, PVS, and bicuspid aortic valve were risk factors for adverse events in the surgical treatment of SVAS [4, 19]. In addition, the mortality rate was lower compared with other centers (3.1–10%) [20, 21, 22]. Therefore, considering the poor preoperative baseline in the pericardium patch group, the pericardium patch performed acceptably in terms of safety.

### 4.3 Effectiveness Assessment

In terms of assessinig effectiveness,, the following characteristics were needed for a good patching material: good histocompatibility and resistance to re-calcification leading to restenosis and sinus deformation, having the potential to grow or not restrict the growth of the aortic root, and being relatively soft to avoid excessive stiffness to squeeze the coronary ostium and cause stenosis, and is sufficient to withstand aortic root pressure [23] and, most importantly, meeting the operating habits of most surgeons. Although the modified patch and the artificial patch have the advantage of high strength, their composite outcome rates (re-operation or restenosis) (modified patch 26.3%; artificial patch 32.4%) were significantly higher than that of the pericardium patch (9.3%) during follow-up.

Based on clinical experience, the pericardial patch could be used for all three surgical methods because of its good pliability and better hemostasis of the suture. Also, subgroup analysis showed that the advantage of the pericardium patch was greater in the two-patch method compared with the single-patch method, possibly suggesting that the single-patch method is associated with a higher incidence of restenosis and reoperation. The disadvantages of the single-patch method have been described in the literature [24]. Therefore, the two-patch method has replaced the single-patch method as the do-often-used technique in our center recently.

Early animal experiments indicated that the pericardium had growth potential, good histocompatibility and its toughness is no less than that of the glutaraldehyde-soaked autologous pericardium [25]. Surgical treatment of the aortic root demonstrated that the aortic sinus structure treated with autologous pericardium was closer to the physiological structure, and had a lower restenosis rate compared with other patches [18]. Hazekamp *et al*. [26] used autologous pericardium in 29 SVAS patients and showed a significant reduction in pressure gradient with no restenosis or aortic regurgitation in all. Cruz-Castañeda *et al*. [27] reported nine cases of autologous pericardium and artificial patch, and found that the pericardium patch had a lower postoperative pressure gradient. These studies suggested that the autologous pericardium could grow with the body after implantation and reduce the occurrence of restenosis. What’s more, the glutaraldehyde-soaked autologous pericardium patch was considered an independent risk factor of restenosis after aortic arch reconstruction [28]. Minakata *et al*. [29] reported eight SVAS pediatric patients using the artificial patch (polyester material), two of whom underwent reoperation for restenosis, and one of whom died. All of the above studies could support our findings.

Our study also found that the pericardium patch had more superiority at the composite outcome in children (age <7 years) and male patients. Children and males have more room to grow at the aortic root, but the modified and artificial patch have no growth potential, limiting the growth of the aortic root, which leads to restenosis. Previous studies [30, 31] indicated a poor long-term prognosis for SVAS pediatric patients using modified pericardium patches, with re-operation rates of 29.7% and 59.2%, respectively. In addition, some studies have demonstrated that male was a risk factor for residual aortic stenosis [32]. Thus, the advantage of the pericardium patch with growth potential is more prominent in children and male patients.

### 4.4 Strengths and Limitations

This study firstly compared the prognosis of the pericardium patch, modified patch, and artificial patch based on a large sample size study and adequate adjustment for possible confounding factors. However, some limitations still exist. First, our study was not a randomized controlled trial or prospective cohort study. Second, information on Williams syndrome was not available because genetic testing of patients was not performed at our center, but we provided detailed concomitant cardiovascular anomalies to avoid bias. Third, the median follow-up time for this data was 24-months, and there was a lack of long-term follow-up results, but we will continue to follow up on our center’s patients to obtain long-term follow-up results.

## 5. Conclusions

The pericardium patches were used most for adolescents (<14 years) SVAS repair in our centers. And, the pericardium patches had lower rates of middle-term reoperation or restenosis along with reliable safety compared with the modified patch and the artificial patch. For children aged <7 years, male, two-patch method, and experienced operator, the pericardium patch showed an obvious superiority.

## Data Availability

The datasets generated during and/or analyzed during the current study are not publicly available due this dataset will continue to be used in subsequent studies but are available from the corresponding author upon reasonable request.

## Abbreviations

BSA, body surface area; ECMO, extracorporeal membrane oxygenation; LVEF, left ventricular ejection function; PVS, pulmonary valve stenosis; STJ, sinotubular junction; SVAS, supravalvular aortic stenosis; TTE, trans-thoracic echocardiogram.

## Supplementary Material

Supplementary material associated with this article can be found, in the online version, at https://doi.org/10.31083/j.rcm2501014.
