# Supplementary material for: Effectiveness and Safety of Different Patch Materials for Supravalvar Aortic Stenosis (Middle-Term Outcomes)
Source: Rev Cardiovasc Med. 2024 Jan 9;25(1):14. doi: 10.31083/j.rcm2501014 (PMC11262331; doi:10.31083/j.rcm2501014)
Supplement: Supplementary file 1 [file 2153-8174-25-1-014-s1.docx]

Effectiveness and Safety of Different Patch Materials for Supravalvar Aortic Stenosis (Middle-Term Outcomes)

(SupplementaryMaterial)

Supplementary Table 1. Concomitant cardiovascular anomalies of patients with SVAS [N (%)].

| Variables | Pericardium patch  (n=133) | Modified patch  (n=43) | Artificial patch  (n=42) | *P* value |
| --- | --- | --- | --- | --- |
| Concomitant cardiovascular anomaly | | | | |
| ACAPA | 2 (1.5) | 0 (0.0) | 0 (0.0) | 0.525 |
| Ascending aortic stenosis | 11 (8.3) | 0 (0.0) | 3 (7.1) | 0.154 |
| PDA | 13 (9.8) | 3 (7.0) | 0 (0.0) | 0.106 |
| VSD | 12 (9.0) | 1 (2.3) | 2 (4.8) | 0.267 |
| TOF | 18 (13.5) | 2 (4.7) | 1 (2.4) | 0.048 |
| HOCM | 1 (0.8) | 0 (0.0) | 0 (0.0) | 0.725 |
| MVS | 1 (0.8) | 0 (0.0) | 0 (0.0) | 0.725 |
| MVR | 2 (1.5) | 0 (0.0) | 0 (0.0) | 0.525 |
| AVS | 4 (3.0) | 0 (0.0) | 0 (0.0) | 0.272 |
| AVR | 13 (9.8) | 3 (7.0) | 3 (7.1) | 0.786 |
| Supra-aortic septum | 26 (19.5) | 6 (14.0) | 5 (11.9) | 0.434 |
| Subaortic septum | 7 (5.3) | 2 (4.7) | 2 (4.8) | 0.983 |

Abbreviation: ACAPA=Anomalous left coronary artery from pulmonary artery; AVR = Aortic valve regurgitation; AVS = Aortic valve stenosis; HOCM= Hypertrophic obstructive cardiomyopathy; MVR = Mitral valve regurgitation; MVS = mitral valve stenosis; PDA = Patent ductus arteriosus; SVAS = Supravalvular aortic stenosis; TOF = Tetralogy of fallot; VSD = Ventricular septal defect.


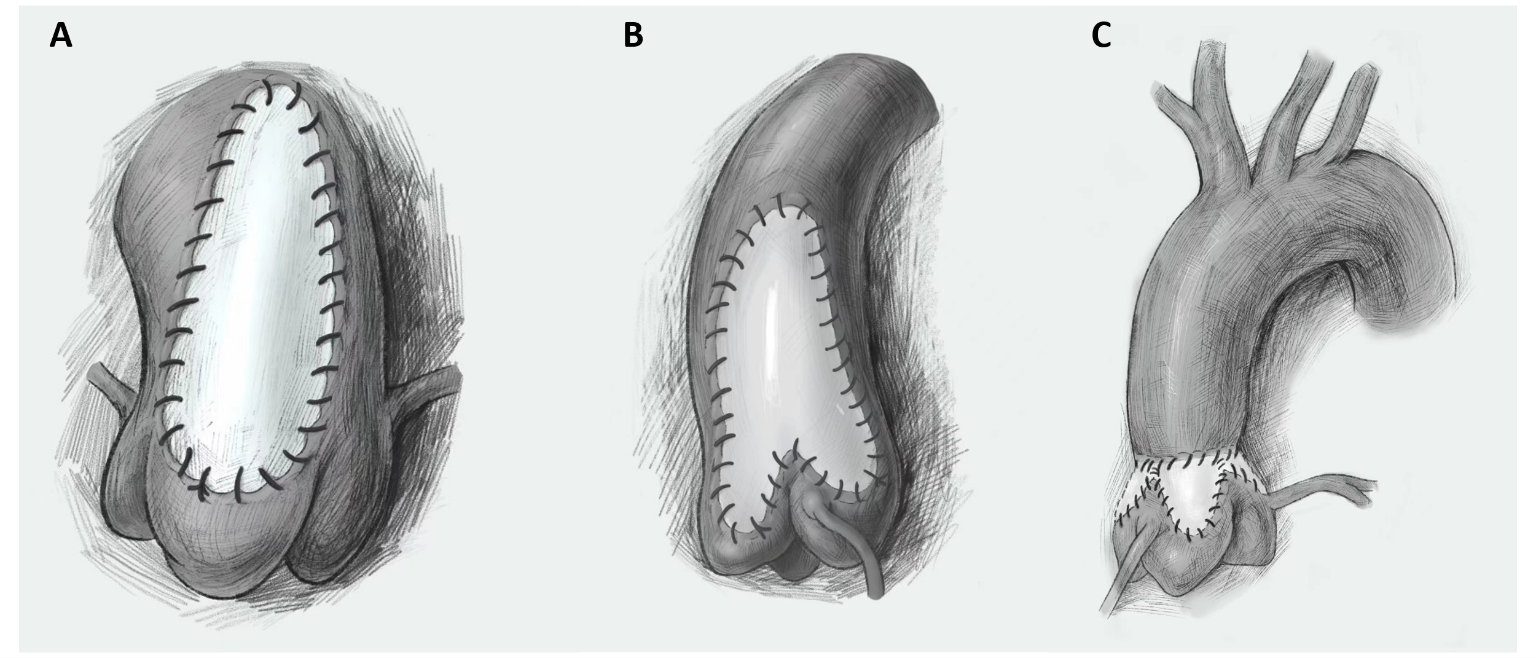


**Supplementary Fig. 1. Three surgical techniques.** A, Single-patch method (McGoon repair). B, Two-patch method (Doty repair) with a pantaloon-shaped patch. C, Three-patch method (Brom repair).

**Supplementary Fig. 2. Patch material was used in hospital from 2002 to 2020 [N (%)].** The left Y-axis was the number of patients and the right Y-axis was the median age (months) per two years of the patients.
